# Coronary Computed Tomography Angiography Atherosclerotic Plaque Volume as A Predictor of Myocardial Blood Flow Impairment in Non-Obstructive Coronary Artery Disease

**DOI:** 10.31083/RCM39291

**Published:** 2025-09-26

**Authors:** Alina N. Maltseva, Raisa V. Dorzhieva, Kristina V. Kopeva, Ayana S. Dasheeva, Andrew V. Mochula, Elena V. Grakova, Konstantin V. Zavadovsky

**Affiliations:** ^1^Cardiology Research Institute, Tomsk National Research Medical Center, Russian Academy of Sciences, 634012 Tomsk, Russia

**Keywords:** atherosclerotic plaque features, non-obstructive coronary artery disease, coronary microvascular dysfunction, myocardial perfusion, myocardial blood flow, myocardial flow reserve, coronary computed tomography angiography, dynamic CZT-SPECT

## Abstract

**Background:**

Studies have demonstrated that patients with non-obstructive coronary artery disease (NOCAD) have an increased risk of myocardial infarction and all-cause mortality, particularly due to coronary microvascular dysfunction (CMD). Moreover, the features of atherosclerotic plaque can affect myocardial blood flow (MBF); however, data on these findings remain limited. Therefore, this study aimed to assess the impact of quantitative coronary computed tomography angiography (CCTA)-derived atherosclerotic plaque features on myocardial perfusion and MBF in NOCAD patients measured using dynamic cadmium–zinc–telluride single-photon emission computed tomography (CZT-SPECT).

**Methods:**

Based on the CCTA results, a total of 49 NOCAD patients (stenosis <50%, 29 men, mean age 57.4 ± 9.0 years) were included in the study. In addition to estimating stenosis severity, the plaque volume (mm^3^) and burden (%) were measured using the coronary bed and separately by structural components (non-calcified, soft-tissue, fibrous, calcified). All patients underwent dynamic CZT-SPECT to assess stress and resting MBF and myocardial flow reserve (MFR).

**Results:**

Based on the MFR values, patients were divided into two groups: Group 1 consisted of patients with reduced MFR (<2.0, n = 20), and Group 2 consisted of those with normal MFR (≥2.0, n = 29). Not all patients had severe myocardial perfusion abnormalities, as determined by standard myocardial perfusion imaging indexes. Analysis of the CCTA data demonstrated that small volumes and burdens of atherosclerotic plaques were characteristic of patients. Stress was significantly correlated with total plaque volume (Spearman's rank correlation coefficient (ρ) = –0.402) and burden (ρ = –0.374), as well as non-calcified plaque volume (ρ = –0.341) and burden (ρ = –0.314). Rest significantly correlated with total plaque volume (ρ = –0.504) and burden (ρ = –0.432), and non-calcified plaque volume (ρ = –0.471) and burden (ρ = –0.433). Meanwhile, MFR and standard indexes of myocardial perfusion impairment did not exhibit significant associations with quantitative CCTA parameters. Multivariate logistic regression analysis revealed that only total plaque volume (odds ratio 1.01; 95% confidence interval 1.005–1.030; *p* < 0.001) was an independent predictor of reduced stress-related MBF of less than 1.5 mL/min/g.

**Conclusions:**

Total plaque volume, derived from quantitative CCTA data, represents an independent predictor of reduced stress-related MBF of less than 1.5 mL/min/g, as obtained using dynamic CZT-SPECT, even in the absence of obstructive coronary artery disease (CAD).

## 1. Introduction

Perspectives on non-obstructive coronary artery disease (NOCAD) have changed over the past decade. The prevalence of patients with NOCAD has been increasing in relation to obstructive coronary artery disease (CAD) [1]. Studies have demonstrated that patients with NOCAD have a high risk of myocardial infarction and all-cause mortality, compared to those without evidence of CAD on coronary computed tomography angiography (CCTA) [2, 3]. Moreover, when the calcified plaque burden is similar, the risk of major adverse cardiovascular events is comparable for both NOCAD and obstructive CAD patients [4]. Thus, the current paradigm of CAD management, which focuses on flow-limiting obstructive stenosis, is imperfect since a relationship exists between myocardial ischemia and coronary atherosclerosis, even in the absence of flow-limiting stenosis [5]. A Working Group for the European Society of Cardiology has suggested that approximately two-thirds of patients with suspected CAD and NOCAD may experience disease progression and the development of complications due to the presence of coronary microvascular dysfunction (CMD) [6]. Additionally, the 2024 European Society of Cardiology guidelines categorize patients with ischemia or angina in the context of NOCAD as a distinct subgroup of chronic coronary syndromes [7]. Consequently, a growing necessity exists to extend beyond anatomical evaluations of the coronary bed and to conduct comprehensive investigations into myocardial microcirculation.

CCTA is a modern, non-invasive tool that can identify coronary stenosis and quantify the extent of plaques. Comprehensive CCTA image processing software enables the assessment of total plaque volume and the burden of different plaque types, including calcified, non-calcified, and low-attenuation plaques [8].

Parameters of CCTA-derived coronary atherosclerosis correlate with myocardial ischemia on gated myocardial perfusion obtained by single-photon emission computed tomography (SPECT) in obstructive CAD patients [9, 10, 11]. Moreover, recent advances in the myocardial dynamic SPECT technique can enable the assessment of myocardial blood flow (MBF) and myocardial flow reserve (MFR) using cadmium–zinc–telluride single-photon emission computed tomography (CZT-SPECT) gamma cameras. The high diagnostic performance of this technique in assessing obstructive CAD and ischemia has been confirmed through the use of invasive coronary angiography, invasive fractional flow reserve (FFR), and positron emission tomography (PET) as reference methods [12, 13, 14, 15, 16]. However, data regarding the use of CZT-SPECT in NOCAD patients to identify those at increased cardiac risk due to CMD remain limited. Moreover, a gap exists in research concerning the associations between CCTA-based plaque quantification and MBF and MFR evaluations in NOCAD patients derived from dynamic CZT-SPECT. Patients with NOCAD who have decreased stress MBF and MFR are at higher risk for CMD and may benefit from additional risk stratification as well as a personalized, modern, and more intensive medication strategy.

Therefore, this study aimed to assess the influence of quantitative CCTA-derived atherosclerotic plaque features on myocardial perfusion and MBF obtained by dynamic CZT-SPECT in NOCAD patients.

## 2. Materials and Methods

### 2.1 Study Design

**Fig. 1.**
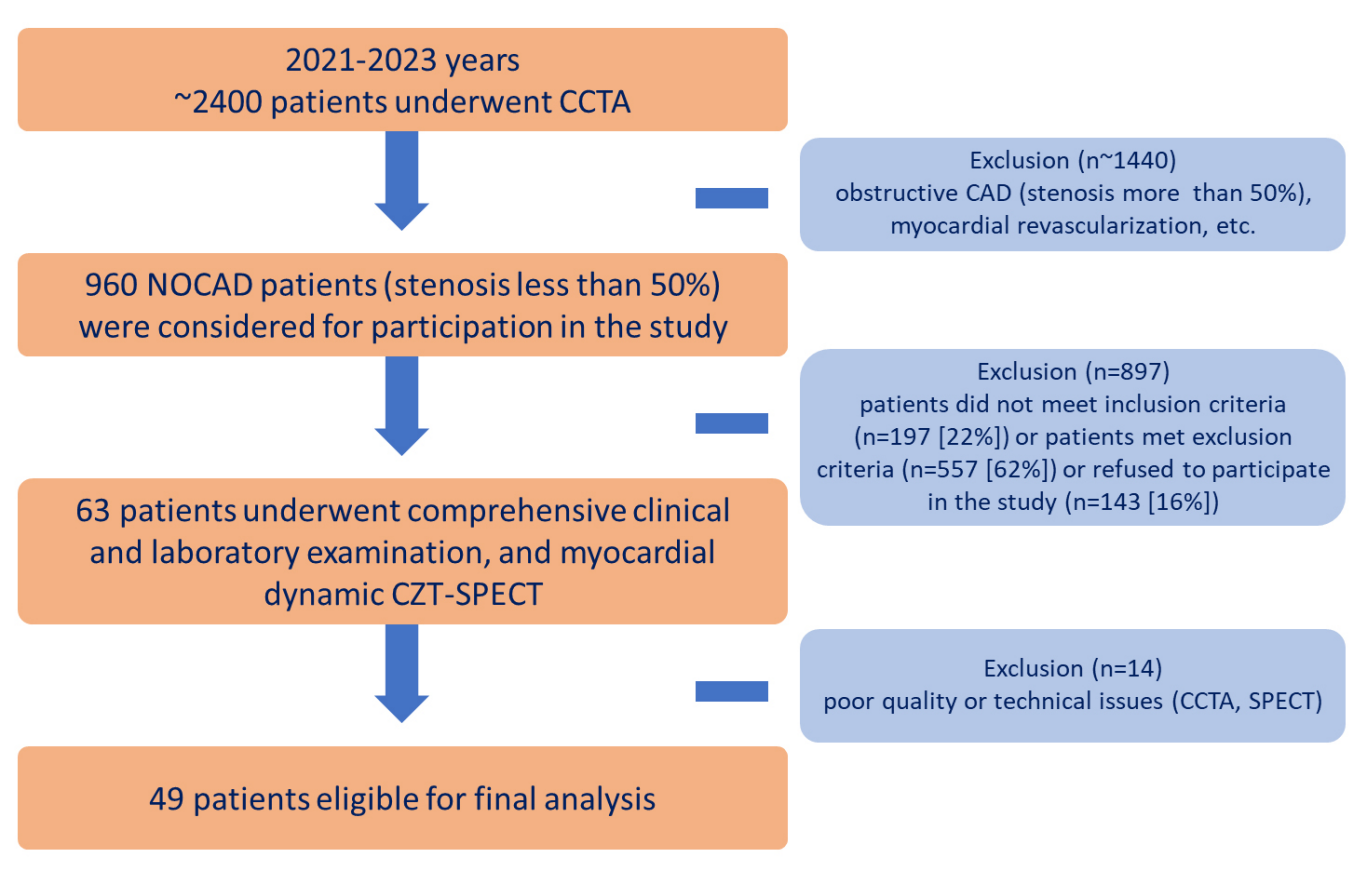
**Flowchart of the study design**. CAD, coronary artery disease; CCTA, coronary computed tomography angiography; CZT, cadmium–zinc–telluride detectors; n, number of patients; NOCAD, non-obstructive coronary artery disease; SPECT, single-photon emission computed tomography.

From 2021 to 2023, 2400 low-risk patients with suspected CAD underwent CCTA according to clinical indications [7, 17, 18]. A total of 960 NOCAD patients (stenosis <50%) were considered for participation in this single-center prospective study. A flowchart of the study design is presented in Fig. 1. The statistical analysis included data from 49 patients; therefore, the study should be considered exploratory in nature.

The inclusion criteria were NOCAD (<50%) confirmed by CCTA, a documented left ventricular (LV) ejection fraction of ≥50%, a sinus rhythm, and the ability to provide signed informed consent. The exclusion criteria were CCTA signs of LV hypertrophy or dilation; previous myocardial infarction or myocardial revascularization; uncontrolled or resistant arterial hypertension; decompensated diabetes mellitus; morbid obesity (body mass index (BMI) ≥40 kg/m^2^); chronic kidney disease stage >2; moderate or severe stenosis and/or insufficiency of the heart valves; cardiomyopathies; ventricular extrasystole ≥3 grades (Lown); atrioventricular block II–III degree or sinus node weakness syndrome; acute and chronic inflammatory heart disease; severe bronchial asthma and/or chronic obstructive pulmonary disease; systemic disease; malignant neoplasms.

A total of 49 patients were enrolled in the final analysis. All patients underwent a comprehensive clinical examination, which included a medical history, blood tests (complete blood count, blood biochemistry test, lipid profile, hemostasis panel), electrocardiography, echocardiography, CCTA, and myocardial dynamic CZT-SPECT perfusion imaging with the assessment of MBF and MFR. Image processing was performed at the Core Facility “Medical Genomics”.

### 2.2 Coronary Computed Tomography Angiography

All CCTA was performed using a hybrid system Discovery NM/CT 570c (GE Healthcare, Milwaukee, WI, USA), equipped with a 64-detector row computed tomography scanner.

A non-enhanced prospective electrocardiographic-gated (ECG) axial scan was triggered at 75% of the R–R interval to measure the coronary artery calcium score. The scanning was performed from the tracheal bifurcation to the level of the diaphragm with breath-holding (6–8 sec) using the following parameters: 2.5 mm slice thickness, 400 ms gantry rotation time, 120 kV tube voltage, 200 mA tube current.

For CCTA, blocker medication was administered (if not contraindicated) with a heart rate >65 beats/min, using intravenous metoprolol (FSUE “Moscow Endocrine Plant”, Russian Federation) at a dosage of 1–10 mg. All patients received sublingual nitroglycerin (LLC “Ozon”, Russian Federation) at a dose of 0.5 mg to improve the visualization of coronary arteries. For the CCTA acquisition, an 80–90 mL bolus of non-ionic iodinated contrast (Ultravist 370, Bayer, Germany) was injected into the antecubital vein at a rate of 5 mL/s, followed by 60 mL of saline at the same rate. The scan range was from 1 cm below the tracheal bifurcation to the level of the diaphragm. The CCTA scan parameters were as follows: 0.35 s gantry rotation time, 64 × 0.625 mm collimation, 25 × 25 cm field of view, 120 kV tube voltage, 450–700 mA tube current. Depending on the heart rate of the patient, a retrospective or prospective ECG was used. The effective radiation dose using the prospective ECG protocol was 1.5–3 mSv, and the retrospective ECG scanning was 15–25 mSv.

Reconstruction of CCTA data was performed at 45% and 75% of the R–R interval using the following parameters: 0.625 mm slice thickness and 0.625 mm interslice interval. Reconstructed images were transferred into a post-processing workstation, Advanced Workstation 4.7 (GE Healthcare, Milwaukee, WI, USA), and were evaluated by two readers blinded to the clinical data. Reconstructed images were assessed by using CardIQ Xpress 2.0 (GE Healthcare, Milwaukee, WI, USA). Reveal software was used for the axial images, curved multiplanar reformations, and cross-sectional analysis, with manual corrections performed as needed.

The calcium score was calculated using SmartScore 4.0 software (GE Healthcare, Milwaukee, WI, USA) according to the method described by Agatston *et al*. [19].

Only segments without significant artefacts were used for further CCTA analyses. Only segments with a diameter of ≥2.0 mm were included. The degree of stenosis (%) was assessed using cross-sectional and curved multiplanar reconstructions.

CCTA data were analyzed using dedicated software, PlaqID, of the CardIQ Xpress 2.0 Package (GE Healthcare, Milwaukee, WI, USA). Automated delineation of the lumen to vessel wall boundaries, as well as vessel wall to perivascular adipose tissue boundaries, was performed, followed by manual adjustment, for each coronary artery equal to or more than 2 mm in diameter. Quantitative plaque analysis was performed at both vessel-based and patient-based levels. The following quantitative variables were calculated: lumen (mm^3^); total volume (mm^3^); total plaque volume (mm^3^); non-calcified plaque volume (as a sum of soft-tissue and fibrous plaque volumes, mm^3^); soft-tissue plaque volume (mm^3^); fibrous plaque volume (mm^3^); calcified plaque volume (mm^3^); total plaque burden (as ratio plaque volume to the sum of lumen volume plus plaque volume × 100, %); non-calcified plaque burden (%); soft-tissue plaque burden (%); fibrous plaque burden (%); calcified plaque burden (%). The atherosclerotic plaque features are defined as follows: soft-tissue plaque, a range of values from -30 to 130 Hounsfield unit (HU); fibrous plaque, a range of values from 131 to 350 HU; calcified plaque, for values greater than 350 HU [20].

### 2.3 Myocardial Dynamic Cadmium-Zinc-Telluride Single-Photon Emission Computed Tomography (Dynamic CZT-SPECT)

The preparation process of the patients, study protocol, data acquisition, and analysis have been previously published [21, 22, 23]. The mean time interval between CCTA and myocardial dynamic CZT-SPECT was 11.1 ± 8.2 days. All myocardial dynamic CZT-SPECTs were performed using a Discovery NM/CT 570c scanner (GE Healthcare, Milwaukee, WI, USA).

Myocardial dynamic CZT-SPECT consisted of two stages of examination: (1) Dynamic recording of the first passage of the radiopharmaceutical bolus through the heart chambers and myocardium to determine quantitative scintigraphic parameters; (2) myocardial perfusion imaging: 60 minutes after the first stage to assess standard indexes of perfusion impairment.

A rest–stress two-day protocol using the radiopharmaceutical agent ^99m^Tc-methoxy-isobutyl-isonitrile (LLC “Diamed”, Russian Federation) was performed in all patients. A pharmacological stress test was performed using adenosine triphosphate (LLC “Ellara”, Russian Federation) infusion at a dosage of 160 mcg/kg/min over 4 minutes [24]. The total effective radiation dose of the study ranged from 4.4 to 7.5 mSv.

All studies were analyzed on the dedicated workstation Xeleris 4.0 (GE Healthcare, Haifa, Israel) using the Corridor 4DM SPECT and 4DM Reserve v.2017 (INVIA, Ann Arbor, MI, USA) software. The one-compartment (single-component) model (1CAC) with attenuation correction was used for quantitative analysis.

Following post-processing, the following quantitative scintigraphic parameters were obtained: stress myocardial blood flow (stress MBF), rest myocardial blood flow (rest MBF), MFR, and flow difference (FD), as well as standard indexes of myocardial perfusion impairment: summed stress score (SSS), summed rest score (SRS), and summed difference score (SDS), as well as parameters of LV contractility: ejection fraction (EF), end diastolic volume (EDV), and end systolic volume (ESV).

Next, patients were divided into two groups based on the MFR values obtained from dynamic CZT-SPECT: (1) reduced MFR <2.0; (2) normal MFR ≥2.0, as based on PET studies [25, 26, 27]. A stress MBF value less than 1.5 mL/min/g was considered abnormal, in conjunction with previous PET and SPECT studies [28, 29].

The local intra- and inter-observer reproducibility of CZT SPECT stress MBF and MFR was estimated in our previous study [29].

### 2.4 Echocardiography

All two-dimensional (2D, B-real-time) transthoracic echocardiography was performed using an ultrasound scanner with enhanced visualization, Philips Affiniti 70 (Philips, Netherlands). The following LV contractility echocardiographic parameters were obtained: EF, EDV, and ESV. A preserved LV systolic function was considered if LV EF ≥50%.

### 2.5 Blood Tests

Patients had blood drawn for testing on the same day as the first stage of dynamic CZT-SPECT. The following blood test parameters were obtained: complete blood count (leukocytes, platelets, hemoglobin), blood biochemistry test (fasting glucose, estimated glomerular filtration rate, C-reactive protein), lipid profile (total cholesterol, low-density lipoprotein cholesterol, high-density lipoprotein cholesterol, triglycerides, non-high-density lipoprotein cholesterol), and hemostasis panel (international normalized ratio, activated partial thromboplastin time, fibrinogen).

### 2.6 Statistical Analysis

Statistical analysis and graphical images were generated using the statistical software R, version 4.3.3 (R Foundation for Statistical Computing, Vienna, Austria), and Jamovi, version 2.2.5.0 (The Jamovi Project, Australia). The distribution of continuous variables was calculated using the Shapiro–Wilk test. Normally distributed continuous variables are presented as the mean value (M) and standard deviation (SD), while non-normally distributed ones are presented as the median (Me) and interquartile range (25th and 75th percentiles). Categorical variables are presented as counts and percentages—n (%). The Mann–Whitney criteria were employed to perform statistical comparisons between the two groups. Categorical variables were compared using the Pearson’s chi-squared test with continuity correction, if necessary, or the Fisher’s exact test. Spearman’s rank correlation coefficient (ρ) was used to analyze rank correlation. Predictors of a reduced stress MBF of less than 1.5 mL/min/g were estimated using univariate and multivariate logistic regression analysis by determining the odds ratio (OR) and 95% confidence interval (CI) [28, 29]. To evaluate intra- and inter-observer reproducibility, a set of 20 patients was assessed independently by two expert radiologists who were blinded to each other’s results. The intraclass correlation coefficient (ICC) was used to quantify reproducibility, with values under 0.50 considered poor, between 0.50 and 0.75 moderate, between 0.75 and 0.90 good, and above 0.90 excellent. In all statistical analyses, a two-tailed *p*-value of 0.05 or less was considered to indicate statistical significance.

## 3. Results

A total of 49 patients (stenosis <50%, 29 men, mean age 57.4 ± 9.0 years) were included in the final analysis. Based on the MFR values obtained from dynamic CZT-SPECT, patients were divided into two groups. The patients enrolled in group 1 exhibited reduced MFR (<2.0, n = 20; 9 men; mean age, 56.3 ± 9.0 years), while the patients in group 2 had normal MFR (≥2.0, n = 29; 20 men; mean age, 58.2 ± 8.9 years).

The clinical and demographic characteristics of the patients did not differ between the groups (Table 1). Since patients were enrolled in the study at the early stages of the disease, these patients did not receive optimal medical therapy at the time of inclusion in the study. The groups did not differ significantly in the frequency of prescribed drugs. Subsequently, the treatment was corrected, and guided-derived medical therapy was prescribed in accordance with current clinical recommendations [7, 17, 18].

**Table 1.** **Clinical and demographic characteristics of NOCAD patients**.

Parameters		Total patients, n = 49	Group 1: reduced MFR (MFR <2.0), n = 20	Group 2: normal MFR (MFR ≥2.0), n = 29	*p*-value
Age, years		57.4 ± 9.0	56.3 ± 9.0	58.2 ± 8.9	0.60
Male sex, n (%)		29 (59%)	9 (45%)	20 (69%)	0.09
Arterial hypertension, n (%)		46 (94%)	19 (95%)	27 (93%)	1.00
Dyslipidemia, n (%)		26 (53%)	11 (55%)	15 (52%)	0.82
Current smoking, n (%)		15 (31%)	4 (20%)	11 (38%)	0.18
BMI, kg/m^2^		29.0 (27.1; 31.6)	29.2 (27.2; 32.1)	29.0 (27.1; 31.2)	0.74
Type 2 diabetes mellitus, n (%)		2 (4%)	1 (5%)	1 (3%)	1.00
Echocardiography data					
	EF, %	65.0 (64.0; 67.0)	65.0 (64.0; 67.3)	65.0 (64.0; 67.0)	0.97
	EDV, mL	107.0 (93.5; 121.0)	102.0 (96.5; 114.0)	112.0 (91.5; 125.0)	0.21
	ESV, mL	37.0 (33.0; 45.0)	35.0 (32.0; 39.5)	41.0 (33.5; 46.0)	0.09
Drug therapy					
	Beta-blockers, n (%)	22 (45%)	9 (45%)	13 (45%)	0.99
	ACE inhibitors, n (%)	16 (33%)	7 (35%)	9 (31%)	0.77
	ARBs, n (%)	10 (20%)	3 (15%)	7 (24%)	0.68
	Diuretics, n (%)	10 (20%)	5 (25%)	5 (17%)	0.76
	Calcium channel blockers, n (%)	14 (29%)	4 (20%)	10 (35%)	0.27
	Antiplatelet agents, n (%)	20 (41%)	9 (45%)	11 (38%)	0.62
	Anticoagulants, n (%)	4 (8%)	0	4 (14%)	0.23
	Lipid-lowering therapy, n (%)	26 (53%)	10 (50%)	16 (55%)	0.72

Table footnotes: All data are presented as the median and interquartile range (IQR), expressed as Me (Q25; Q75), as the mean ± SD, or as n (%). ACE inhibitors, angiotensin-converting enzyme inhibitors; ARBs, angiotensin II receptor blockers; BMI, body mass index; EDV, end diastolic volume; EF, ejection fraction; ESV, end systolic volume; MFR, myocardial flow reserve; n, number of patients; NOCAD, non-obstructive coronary artery disease.

Patients with reduced MFR exhibited a significantly lower level of activated partial thromboplastin time compared to those with normal MFR. Other blood parameters were found to be negligible (Table 2).

**Table 2.** **Blood tests of NOCAD patients**.

Parameters	Total patients, n = 49	Group 1: reduced MFR (MFR <2.0), n = 20	Group 2: normal MFR (MFR ≥2.0), n = 29	*p*-value
TC, mmol/L	4.70 (3.94; 5.99)	5.18 (4.21; 6.01)	4.62 (3.86; 5.34)	0.36
LDL-C, mmol/L	2.80 (1.80; 3.61)	2.85 (1.80; 3.72)	2.80 (1.80; 3.50)	0.51
HDL-C, mmol/L	1.31 (1.13; 1.59)	1.33 (1.21; 1.71)	1.30 (1.03; 1.54)	0.24
TG, mmol/L	1.60 (0.92; 2.22)	1.70 (0.91; 2.16)	1.58 (1.10; 2.24)	0.98
Non-HDL-C, mmol/L	3.56 (2.42; 4.54)	3.55 (2.73; 4.59)	3.56 (2.16; 4.31)	0.54
Fasting glucose, mmol/L	5.88 (5.49; 6.22)	5.74 (5.38; 6.09)	6.03 (5.56; 6.30)	0.08
eGFR, mL/min/1.73 m^2^	76.20 (67.90; 87.20)	82.00 (67.20; 96.50)	75.30 (67.90; 80.20)	0.20
CRP, mg/L	3.10 (2.50; 4.00)	3.00 (2.50; 3.90)	3.30 (2.50; 3.80)	0.77
Leukocytes, 10^9^/L	6.30 (5.47; 7.13)	6.19 (5.48; 7.11)	6.40 (5.50; 7.13)	0.98
Platelets, 10^9^/L	244.00 (195.00; 291.00)	250.00 (207.00; 298.00)	225.00 (193.00; 271.00)	0.54
Hemoglobin, g/L	144.00 (132.00; 153.00)	144.00 (130.00; 152.00)	142.00 (135.00; 153.00)	0.80
INR	0.95 (0.91; 1.00)	0.96 (0.92; 0.98)	0.94 (0.92; 1.02)	0.90
APTT, sec	27.50 (26.10; 30.30)	26.30 (25.50; 28.80)	28.80 (26.90; 30.80)	0.03
Fibrinogen, g/L	2.92 (2.63; 3.34)	2.90 (2.59; 3.45)	2.92 (2.64; 3.29)	0.85

Table footnotes: All data are presented as the median and interquartile range (IQR), expressed as Me (Q25; Q75). APTT, activated partial thromboplastin time; CRP, C-reactive protein; eGFR, estimated glomerular filtration rate; HDL-C, high-density lipoprotein cholesterol; INR, international normalized ratio; LDL-C, low-density lipoprotein cholesterol; MFR, myocardial flow reserve; n, number of patients; NOCAD, non-obstructive coronary artery disease; non-HDL-C, non-high-density lipoprotein cholesterol; TC, total cholesterol; TG, triglycerides.

The dynamic CZT-SPECT results are presented in Table 3. Patients did not exhibit severe myocardial perfusion abnormalities, as determined by standard myocardial perfusion imaging indexes. Patients with reduced MFR had significantly lower stress MBF and FD, and higher rest MBF compared to the group with normal MFR.

**Table 3.** **Myocardial dynamic CZT-SPECT parameters of NOCAD patients**.

Parameters		Total patients, n = 49	Group 1: reduced MFR (MFR <2.0), n = 20	Group 2: normal MFR (MFR ≥2.0), n = 29	*p*-value
Standard indexes of myocardial perfusion imaging					
	SSS	1.0 (0.0; 2.0)	1.5 (0.0; 3.0)	0.0 (0.0; 2.0)	0.24
	SRS	0.0 (0.0; 0.0)	0.0 (0.0; 0.0)	0.0 (0.0; 0.0)	0.40
	SDS	0.0 (0.0; 2.0)	1.5 (0.0; 3.0)	0.0 (0.0; 2.0)	0.22
Myocardial blood flow and reserve parameters					
	Stress MBF, mL/min/g	1.59 (1.28; 1.93)	1.48 (1.13; 1.68)	1.65 (1.42; 2.21)	0.042
	Rest MBF, mL/min/g	0.76 (0.56; 0.95)	1.01 (0.82; 1.14)	0.62 (0.44; 0.77)	<0.001
	FD, mL/min/g	0.81 (0.47; 1.21)	0.43 (0.15; 0.65)	1.02 (0.88; 1.43)	<0.001

Table footnotes: All data are presented as the median and interquartile range (IQR), expressed as Me (Q25; Q75). CZT, cadmium–zinc–telluride detectors; FD, flow difference; MBF, myocardial blood flow; MFR, myocardial flow reserve; n, number of patients; NOCAD, non-obstructive coronary artery disease; SDS, summed difference score; SPECT, single-photon emission computed tomography; SRS, summed rest score; SSS, summed stress score.

Analysis of the CCTA data demonstrated that patients were characterized by small atherosclerotic plaque volumes and burdens, which aligns with data reported in a large international cohort study [30]. Patients with a normal MFR presented only significantly higher calcium scores (Table 4). The study utilized exclusively coronary atherosclerosis volume and burden parameters, demonstrating good (0.75 < ICC < 0.90) to excellent (ICC >0.90) intra- and inter-observer reproducibility (**Supplementary Table 1**).

**Table 4.** **Coronary computed tomography angiography of NOCAD patients**.

Parameters	Total patients, n = 49	Group 1: reduced MFR (MFR <2.0), n = 20	Group 2: normal MFR (MFR ≥2.0), n = 29	*p*-value
Maximum stenosis, %	35.0 (20.0; 40.0)	32.5 (20.0; 46.3)	35.0 (25.0; 40.0)	0.91
Calcium score, Agatston units	19.0 (0.0; 68.0)	0.0 (0.0; 62.0)	30.0 (4.0; 102.0)	0.02
Lumen, mm^3^	1924.0 (1366.0; 2351.0)	1657.0 (1419.0; 2101.0)	1945.0 (1375.0; 2397.0)	0.45
General volume, mm^3^	1938.0 (1509.0; 2418.0)	1685.0 (1555.0; 2266.0)	2186.0 (1490.0; 2471.0)	0.34
Total plaque volume, mm^3^	51.9 (0.0; 137.0)	13.6 (0.0; 131.0)	68.3 (31.2; 135.0)	0.10
Total plaque burden, %	2.9 (0.0; 7.0)	0.6 (0.0; 6.6)	3.8 (1.3; 7.2)	0.18
Non-calcified plaque volume, mm^3^	36.3 (0.0; 132.0)	16.7 (0.0; 104.0)	49.6 (27.9; 134.0)	0.08
Non-calcified plaque burden, %	2.3 (0.25; 5.9)	0.6 (0.0; 4.8)	3.8 (1.6; 6.4)	0.07
Soft tissue plaque volume, mm^3^	0.1 (0.0; 10.7)	0.0 (0.0; 5.0)	1.6 (0.0; 23.2)	0.09
Soft tissue plaque burden, %	0.01 (0.0; 0.5)	0.0 (0.0; 0.1)	0.1 (0.0; 0.7)	0.07
Fibrous plaque volume, mm^3^	34.3 (0.0; 110.0)	9.4 (0.0; 91.5)	38.4 (27.8; 114.0)	0.08
Fibrous plaque burden, %	2.0 (0.0; 5.2)	0.4 (0.0; 4.2)	2.6 (1.2; 5.2)	0.10
Calcified plaque volume, mm^3^	3.5 (0.0; 19.0)	1.5 (0.0; 17.8)	3.7 (0.6; 17.1)	0.47
Calcified plaque burden, %	0.2 (0.0; 0.9)	0.1 (0.0; 0.8)	0.2 (0.03; 0.8)	0.60

Table footnotes: All data are presented as the median and interquartile range (IQR), expressed as Me (Q25; Q75). MFR, myocardial flow reserve; n, number of patients; NOCAD, non-obstructive coronary artery disease.

**Fig. 2.**
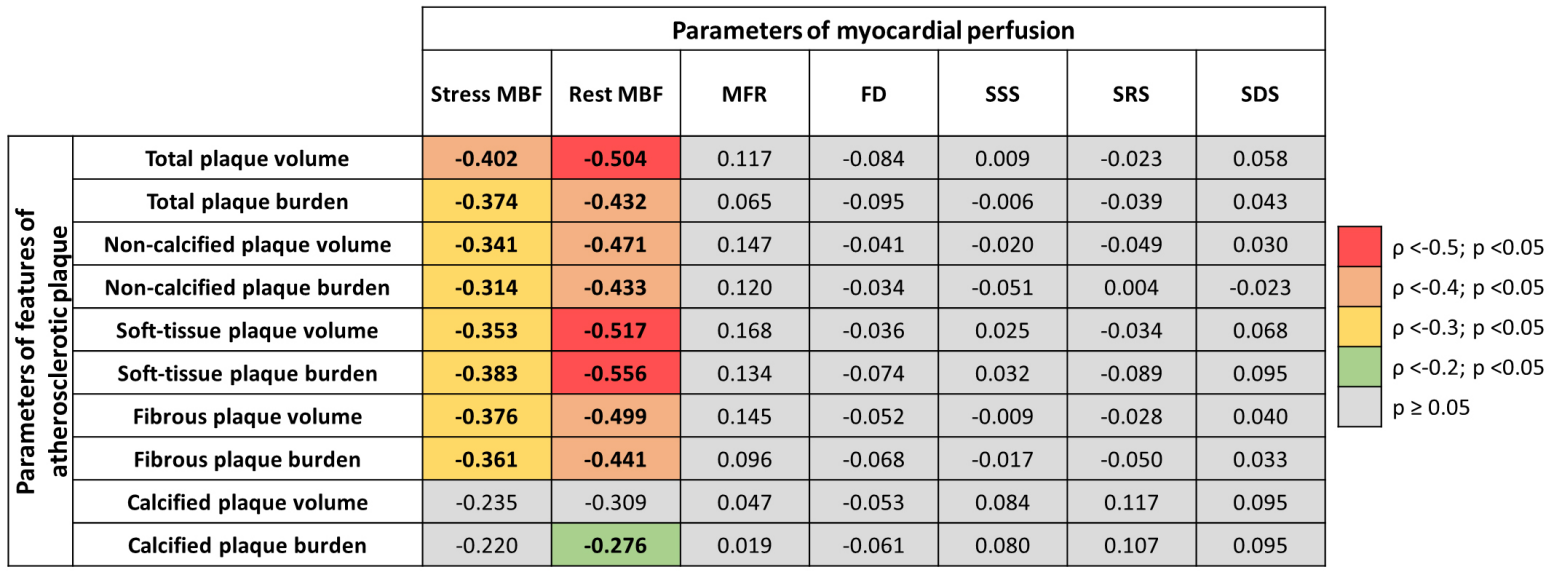
**A heatmap graph of associations between atherosclerotic plaque features and myocardial blood flow in NOCAD patients**. ρ, the Spearman’s correlation coefficient; FD, flow difference; MBF, myocardial blood flow; MFR, myocardial flow reserve; SDS, summed difference score; SRS, summed rest score; SSS, summed stress score.

Stress and rest MBF values are associated with total and non-calcified (soft-tissue and fibrous) plaque volume and burden. Interestingly, MFR, FD, and standard indexes of myocardial perfusion impairment were not significantly associated with quantitative CCTA parameters. The heatmap graph of associations regarding atherosclerotic plaque features and MBF in NOCAD patients is shown in Fig. 2.

Based on the univariate logistic regression analysis results, the following quantitative CCTA parameters were predictors of reduced stress MBF of less than 1.5 mL/min/g: total plaque volume (OR 1.01; 95% CI 1.00–1.02; *p* = 0.006); total plaque burden (OR 1.21; 95% CI 1.05–1.40; *p* = 0.009); non-calcified plaque volume (OR 1.01; 95% CI 1.00–1.02; *p* = 0.007); non-calcified plaque burden (OR 1.22; 95% CI 1.04–1.43; *p* = 0.013); soft-tissue plaque volume (OR 1.07; 95% CI 1.01–0.12; *p* = 0.018); fibrous plaque volume (OR 1.01; 95% CI 1.00–1.02; *p* = 0.008); fibrous plaque burden (OR 1.27; 95% CI 1.06–1.52; *p* = 0.009). Additional covariates, such as age, sex, risk factors, laboratory markers of lipid profile, complete blood count, blood biochemistry tests, hemostasis panel, echocardiographic parameters of left ventricular contractility, and other coronary computed tomography angiography characteristics, were not significant predictors of a reduced stress MBF of less than 1.5 mL/min/g (**Supplementary Table 2**). Based on the stepwise multivariate logistic regression analysis result, adjusted for age, sex, and aforementioned clinical, laboratory, and echocardiographic characteristics, only total plaque volume (OR 1.01; 95% CI 1.005–1.030; *p* < 0.001; Nagelkerke R^2^ = 0.505) was found to be an independent predictor of a reduced stress MBF of less than 1.5 mL/min/g.

**Fig. 3.**
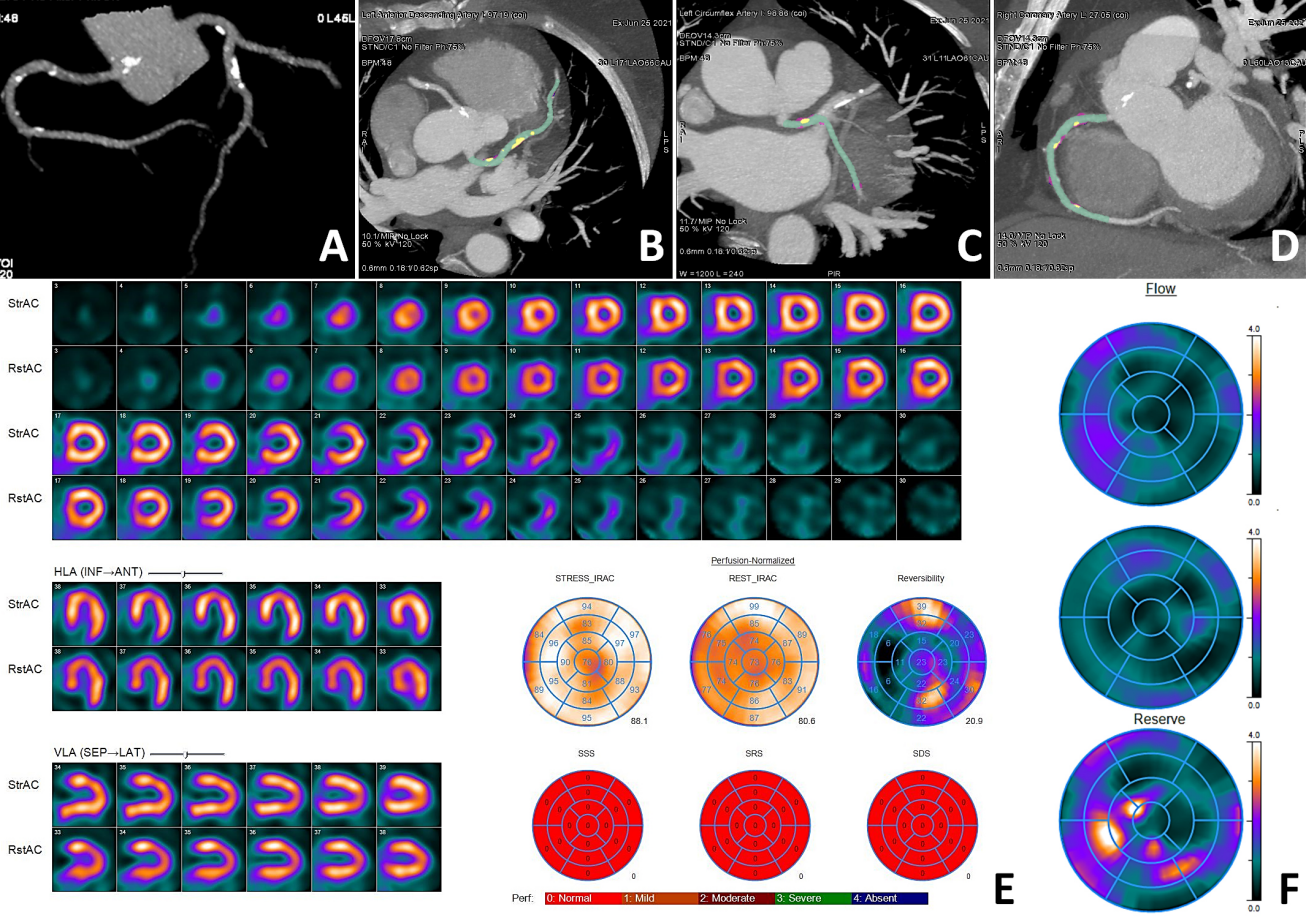
**Clinical case of a NOCAD patient with reduced stress MBF**. Patient K., female, 56 years old, arterial hypertension, dyslipidemia. CCTA ((A) three-dimensional reconstruction of the coronary tree; (B) MIP of the LAD; (C) MIP of the LCX; (D) MIP of the RCA): diffuse NOCAD; maximum stenosis 40% in the proximal segment of the LAD; total plaque volume 288.2 mm^3^; total plaque burden 15.2%; non-calcified plaque volume 239.1 mm^3^; non-calcified plaque burden 12.6%; soft tissue plaque volume 9.5 mm^3^; soft tissue plaque burden 0.5%; fibrous plaque volume 229.6 mm^3^; fibrous plaque burden 12.1%; calcified plaque volume 49.1 mm^3^; calcified plaque burden 2.6%. MPI (E): normal myocardial perfusion; SSS 0; SRS 0; SDS 0. Myocardial dynamic CZT-SPECT (F): reduced stress MBF 0.91 mL/min/g, rest MBF 0.74 mL/min/g, reduced MFR 1.23, FD 0.17 mL/min/g. Green indicates the lumen of the coronary arteries, yellow indicates the calcified structural components of the plaque, and purple indicates the fibrous structural components of the plaque. CAD, coronary artery disease; CCTA, coronary computed tomography angiography; CZT, cadmium–zinc–telluride detectors; FD, flow difference; LAD, left anterior descending artery; LCX, left circumflex artery; MBF, myocardial blood flow; MIP, maximum intensity projection; MFR, myocardial flow reserve; MPI, myocardial perfusion imaging; NOCAD, non-obstructive coronary artery disease; RCA, right coronary artery; SDS, summed difference score; SPECT, single-photon emission computed tomography; SRS, summed rest score; SSS, summed stress score.

The representative clinical case of the NOCAD patient with reduced stress MBF is shown in Fig. 3.

## 4. Discussion

The results of this exploratory study support the notion that CCTA-derived total plaque volume is an independent predictor of a reduced stress MBF of less than 1.5 mL/min/g obtained by dynamic CZT-SPECT, even in the absence of obstructive CAD. Non-calcified (soft-tissue and fibrous) plaque volume and burden were linked with impaired MBF compared with calcified components. These findings support the concept that plaque volume and burden cause myocardial perfusion abnormalities to a greater extent than the degree of stenosis. Moreover, non-calcified plaques, in contrast to calcified ones, were associated with more severe myocardial perfusion abnormalities. These findings are significant in the context of developing an effective treatment strategy and improving prognoses.

Several studies have shown that the influence of stenosis severity on MBF may not be certain [4, 31, 32]. For instance, Schuijf *et al*. [31] reported that 15% of the patients with no stenosis ≥50% assessed by CCTA had perfusion abnormalities in the SPECT analysis. Vattay *et al*. [32] utilized a segment-based analysis to investigate the associations between quantitative plaque characteristics derived from CCTA and segmental myocardial ischemia using dynamic perfusion computed tomography. The authors demonstrated that an increase in total plaque volume was associated with a reduction in MBF, even after adjusting for clinical risk factors, luminal area stenosis, and high-risk plaque characteristics. Moreover, lumen area stenosis and the presence of high-risk plaques were not linked to ischemia. Our findings align with these previous results, as we reported that certain plaque characteristics may be associated with MBF impairment, even in the absence of CAD. However, semiquantitative myocardial perfusion parameters did not show such associations. These results may be explained by endothelial dysfunction, a mechanism currently considered a key contributor to CMD. This leads to impaired nitric oxide release, making it impossible to achieve vasodilation and decreasing MBF [33]. Recent research has shown that morphological atherosclerotic plaque features, particularly the necrotic core volume, can affect MBF and potentially lead to ischemia [34]. In addition, the morphological structure of plaques leads to impairment of FFR regardless of the severity of luminal stenosis [34].

The non-calcified plaque contains a significant amount of cholesterol, making the plaque susceptible to rupture and potentially leading to cardiac events [35]. Recent studies have reported that components in an atherosclerotic plaque, such as lipid necrotic core, low-density non-calcified plaque, may predict myocardial ischemia or decrease MBF [12, 36, 37]. Diaz-Zamudio *et al*. [36] demonstrated this in a cohort of 184 patients with suspected CAD who underwent hybrid CCTA and stress and rest myocardial perfusion imaging scans on the CZT camera.

A low-density non-calcified plaque burden has been associated with ischemia in vessels with stenosis of 30–69% [36]. A similar result has been reported with FFR derived from CCTA for the detection of myocardial ischemia. Gaur *et al.* [37] found that a low-density non-calcified plaque volume of ≥30 mm^3^ predicted ischemia independently of other plaque characteristics and stenosis severity. Wang *et al*. [12] demonstrated that necrotic core volume on the CCTA singly anticipated myocardial ischemia on PET, beyond diameter stenosis alone. Driessen *et al*. [38] examined 208 patients with suspected CAD who underwent CCTA and PET myocardial perfusion imaging. Morphological features such as non-calcified plaque volume and positive remodeling predicted impaired hyperemic MBF and FFR, independent of lesion severity. Indeed, atherosclerotic plaques significantly affect MBF by altering the coronary artery function and structure. The first mechanism is that plaques can directly cause anatomical obstruction of the coronary arteries, and the second mechanism is related to plaque morphology and progression. Thus, coronary atherosclerosis is influenced by CMD, even in the early stages of CAD. Atherosclerotic plaque features might be a pathogenetic link in the development of vascular inflammation and endothelial dysfunction, which may lead to severe myocardial ischemia in the future [38].

Interestingly, the current study revealed no correlation between calcified plaque volume and MBF. This finding aligns with the results of Feuchtner *et al*. [39], who investigated 106 patients with NOCAD undergoing both CCTA and FFR. The authors revealed that an increasing plaque density, in particular calcified lesions, is associated with declining ischemia. Although it is well known that the influence of calcification on the plaque can be different, namely, leading to stability or rupture of the plaque. Wu *et al*. [40] described the mechanisms through which calcifications may play a positive role in plaque stability, and their negative effect depends on the localization of calcifications within the plaque. This finding warrants further investigation in future studies.

## 5. Limitations

The main limitations of this study stem from the single-center design and relatively small sample size, which limit the diversity of patients and the spectrum of disease severity, reduce statistical power, and increase the risk of type II errors—potentially affecting the generalizability of the findings. Thus, this study can be considered preliminary and hypothesis-generating. Larger, multi-center studies with more heterogeneous populations are needed to validate and generalize these findings.

## 6. Conclusions

Total plaque volume, derived from quantitative CCTA data, is an independent predictor of a reduced stress MBF of less than 1.5 mL/min/g obtained by dynamic CZT-SPECT, even in the absence of obstructive CAD. These findings suggest that total plaque volume may serve as an early marker of CMD related to atherosclerosis in NOCAD patients, potentially identifying those at increased risk of developing CMD. The combined quantitative assessment of CCTA parameters and stress MBF could enhance the stratification of cardiovascular risk in the NOCAD population. Nonetheless, further studies are needed to obtain more data related to CZT MBF assessment, which could lead to the development of a model for early and accurate risk assessment of clinical status, treatment dynamics, and the likelihood of major adverse cardiovascular events.

## Data Availability

The datasets used and analyzed during the current study are available from the corresponding author on reasonable request.

## Abbreviations

1CAC, the one-compartment (single-component) model; CAD, coronary artery disease; CI, 95% confidence interval; CMD, coronary microvascular dysfunction; CCTA, coronary computed tomography angiography; CZT, cadmium-zinc-telluride; ECG, electrocardiographic-gated; EDV, end diastolic volume; EF, ejection fraction; ESV, end systolic volume; FD, flow difference; FFR, fractional flow reserve; ICC, intraclass correlation coefficient; LV, left ventricular; MBF, myocardial blood flow; MFR, myocardial flow reserve; NOCAD, non-obstructive coronary artery disease; OR, odds ratio; PET, positron emission tomography; SDS, summed difference score; SPECT, single-photon emission computed tomography; SRS, summed rest score; SSS, summed stress score.

## Supplementary Material

Supplementary material associated with this article can be found, in the online version, at https://doi.org/10.31083/RCM39291.

## References

[1] Hansen B, Holtzman JN, Juszczynski C, Khan N, Kaur G, Varma B (2023). Ischemia with No Obstructive Arteries (INOCA): A Review of the Prevalence, Diagnosis and Management. *Current Problems in Cardiology*.

[2] Min JK, Dunning A, Lin FY, Achenbach S, Al-Mallah M, Budoff MJ (2011). Age- and sex-related differences in all-cause mortality risk based on coronary computed tomography angiography findings results from the International Multicenter CONFIRM (Coronary CT Angiography Evaluation for Clinical Outcomes: An International Multicenter Registry) of 23,854 patients without known coronary artery disease. *Journal of the American College of Cardiology*.

[3] Maddox TM, Stanislawski MA, Grunwald GK, Bradley SM, Ho PM, Tsai TT (2014). Nonobstructive coronary artery disease and risk of myocardial infarction. *JAMA*.

[4] Mortensen MB, Dzaye O, Steffensen FH, Bøtker HE, Jensen JM, Rønnow Sand NP (2020). Impact of Plaque Burden Versus Stenosis on Ischemic Events in Patients With Coronary Atherosclerosis. *Journal of the American College of Cardiology*.

[5] Schuijf JD, Matheson MB, Ostovaneh MR, Arbab-Zadeh A, Kofoed KF, Scholte AJHA (2020). Ischemia and No Obstructive Stenosis (INOCA) at CT Angiography, CT Myocardial Perfusion, Invasive Coronary Angiography, and SPECT: The CORE320 Study. *Radiology*.

[6] Padro T, Manfrini O, Bugiardini R, Canty J, Cenko E, De Luca G (2020). ESC Working Group on Coronary Pathophysiology and Microcirculation position paper on ’coronary microvascular dysfunction in cardiovascular disease’. *Cardiovascular Research*.

[7] Vrints C, Andreotti F, Koskinas KC, Rossello X, Adamo M, Ainslie J (2024). 2024 ESC Guidelines for the management of chronic coronary syndromes. *European Heart Journal*.

[8] Nieman K, García-García HM, Hideo-Kajita A, Collet C, Dey D, Pugliese F (2024). Standards for quantitative assessments by coronary computed tomography angiography (CCTA): An expert consensus document of the society of cardiovascular computed tomography (SCCT). *Journal of Cardiovascular Computed Tomography*.

[9] de Graaf MA, El-Naggar HM, Boogers MJ, Veltman CE, Broersen A, Kitslaar PH (2013). Automated quantitative coronary computed tomography correlates of myocardial ischaemia on gated myocardial perfusion SPECT. *European Journal of Nuclear Medicine and Molecular Imaging*.

[10] Eskerud I, Gerdts E, Larsen TH, Simon J, Maurovich-Horvat P, Lønnebakken MT (2021). Total coronary atherosclerotic plaque burden is associated with myocardial ischemia in non-obstructive coronary artery disease. *International Journal of Cardiology. Heart & Vasculature*.

[11] Tamarappoo BK, Gutstein A, Cheng VY, Nakazato R, Gransar H, Dey D (2010). Assessment of the relationship between stenosis severity and distribution of coronary artery stenoses on multislice computed tomographic angiography and myocardial ischemia detected by single photon emission computed tomography. *Journal of Nuclear Cardiology: Official Publication of the American Society of Nuclear Cardiology*.

[12] Wang X, van den Hoogen IJ, Butcher SC, Kuneman JH, de Graaf MA, Kamperidis V (2023). Importance of plaque volume and composition for the prediction of myocardial ischaemia using sequential coronary computed tomography angiography/positron emission tomography imaging. *European Heart Journal. Cardiovascular Imaging*.

[13] Ben Bouallègue F, Roubille F, Lattuca B, Cung TT, Macia JC, Gervasoni R (2015). SPECT Myocardial Perfusion Reserve in Patients with Multivessel Coronary Disease: Correlation with Angiographic Findings and Invasive Fractional Flow Reserve Measurements. *Journal of Nuclear Medicine: Official Publication, Society of Nuclear Medicine*.

[14] Han S, Kim YH, Ahn JM, Kang SJ, Oh JS, Shin E (2018). Feasibility of dynamic stress 201Tl/rest 99mTc-tetrofosmin single photon emission computed tomography for quantification of myocardial perfusion reserve in patients with stable coronary artery disease. *European Journal of Nuclear Medicine and Molecular Imaging*.

[15] Giubbini R, Bertoli M, Durmo R, Bonacina M, Peli A, Faggiano I (2021). Comparison between N13NH3-PET and 99mTc-Tetrofosmin-CZT SPECT in the evaluation of absolute myocardial blood flow and flow reserve. *Journal of Nuclear Cardiology: Official Publication of the American Society of Nuclear Cardiology*.

[16] Wells RG, Marvin B, Poirier M, Renaud J, deKemp RA, Ruddy TD (2017). Optimization of SPECT Measurement of Myocardial Blood Flow with Corrections for Attenuation, Motion, and Blood Binding Compared with PET. *Journal of Nuclear Medicine: Official Publication, Society of Nuclear Medicine*.

[17] Virani SS, Newby LK, Arnold SV, Bittner V, Brewer LC, Demeter SH (2023). 2023 AHA/ACC/ACCP/ASPC/NLA/PCNA Guideline for the Management of Patients With Chronic Coronary Disease: A Report of the American Heart Association/American College of Cardiology Joint Committee on Clinical Practice Guidelines. *Circulation*.

[18] Barbarash OL, Karpov YuA, Panov AV, Akchurin RS, Alekyan BG, Alekhin MN (2024). 2024 Clinical practice guidelines for Stable coronary artery disease. *Russian Journal of Cardiology*.

[19] Agatston AS, Janowitz WR, Hildner FJ, Zusmer NR, Viamonte M, Detrano R (1990). Quantification of coronary artery calcium using ultrafast computed tomography. *Journal of the American College of Cardiology*.

[20] Shaw LJ, Blankstein R, Bax JJ, Ferencik M, Bittencourt MS, Min JK (2021). Society of Cardiovascular Computed Tomography / North American Society of Cardiovascular Imaging - Expert Consensus Document on Coronary CT Imaging of Atherosclerotic Plaque. *Journal of Cardiovascular Computed Tomography*.

[21] Mochula A, Maltseva A, Kopeva K, Grakova E, Mochula O, Zavadovsky K (2024). The Influence of Kinetic Models and Attenuation Correction on Cadmium-Zinc-Telluride Single-Photon Emission Computed Tomography (CZT SPECT)-Derived Myocardial Blood Flow and Reserve: Correlation with Invasive Angiography Data. *Journal of Clinical Medicine*.

[22] Mochula AV, Kopeva KV, Maltseva AN, Grakova EV, Gulya M, Smorgon AV (2023). The myocardial flow reserve in patients with heart failure with preserved ejection fraction. *Heart and Vessels*.

[23] Mochula AV, Mochula OV, Maltseva AN, Suleymanova AS, Kapilevich NA, Ryabov VV (2023). Quantitative assessment of myocardial blood fl ow by dynamic single photon emission computed tomography: relationship with ECG changes and biochemical markers of damage in patients with acute myocardial infarction. *The Siberian Journal of Clinical and Experimental Medicine*.

[24] Henzlova MJ, Cerqueira MD, Mahmarian JJ, Yao SS, Quality Assurance Committee of the American Society of Nuclear Cardiology (2006). Stress protocols and tracers. *Journal of Nuclear Cardiology: Official Publication of the American Society of Nuclear Cardiology*.

[25] Murthy VL, Naya M, Foster CR, Hainer J, Gaber M, Di Carli G (2011). Improved cardiac risk assessment with noninvasive measures of coronary flow reserve. *Circulation*.

[26] Aljizeeri A, Ahmed AI, Alfaris MA, Ahmed D, Farea J, Elneama A (2021). Myocardial Flow Reserve and Coronary Calcification in Prognosis of Patients With Suspected Coronary Artery Disease. *JACC. Cardiovascular Imaging*.

[27] Patel KK, Peri-Okonny PA, Qarajeh R, Patel FS, Sperry BW, McGhie AI (2022). Prognostic Relationship Between Coronary Artery Calcium Score, Perfusion Defects, and Myocardial Blood Flow Reserve in Patients With Suspected Coronary Artery Disease. *Circulation. Cardiovascular Imaging*.

[28] Bateman TM, Heller GV, Beanlands R, Calnon DA, Case J, deKemp R (2021). Practical Guide for Interpreting and Reporting Cardiac PET Measurements of Myocardial Blood Flow: An Information Statement from the American Society of Nuclear Cardiology, and the Society of Nuclear Medicine and Molecular Imaging. *Journal of Nuclear Medicine: Official Publication, Society of Nuclear Medicine*.

[29] Zavadovsky KV, Mochula AV, Maltseva AN, Boshchenko AA, Baev AE, Andreev SL (2022). The diagnostic value of SPECT CZT quantitative myocardial blood flow in high-risk patients. *Journal of Nuclear Cardiology: Official Publication of the American Society of Nuclear Cardiology*.

[30] Tzimas G, Gulsin GS, Everett RJ, Akodad M, Meier D, Sewnarain K (2024). Age- and Sex-Specific Nomographic CT Quantitative Plaque Data From a Large International Cohort. *JACC. Cardiovascular Imaging*.

[31] Schuijf JD, Wijns W, Jukema JW, Atsma DE, de Roos A, Lamb HJ (2006). Relationship between noninvasive coronary angiography with multi-slice computed tomography and myocardial perfusion imaging. *Journal of the American College of Cardiology*.

[32] Vattay B, Borzsák S, Boussoussou M, Vecsey-Nagy M, Jermendy ÁL, Suhai FI (2022). Association between coronary plaque volume and myocardial ischemia detected by dynamic perfusion CT imaging. *Frontiers in Cardiovascular Medicine*.

[33] Sakr SA, Abbas TM, Amer MZ, Dawood EM, El-Shahat N, Abdel Aal IA (2009). Microvascular angina. The possible role of inflammation, uric acid, and endothelial dysfunction. *International Heart Journal*.

[34] Ahmadi A, Senoner T, Correa A, Feuchtner G, Narula J (2020). How atherosclerosis defines ischemia: Atherosclerosis quantification and characterization as a method for determining ischemia. *Journal of Cardiovascular Computed Tomography*.

[35] Pour-Ghaz I, Alkhatib D, Isa S, Al-Taweel O, Ugonabo I, Yedlapati N (2023). The Roles of Coronary Computed Tomography Angiography in Characterizing Coronary Plaque: Screening, Treatment, and Prevention. *Journal of Cardiovascular Development and Disease*.

[36] Diaz-Zamudio M, Fuchs TA, Slomka P, Otaki Y, Arsanjani R, Gransar H (2017). Quantitative plaque features from coronary computed tomography angiography to identify regional ischemia by myocardial perfusion imaging. *European Heart Journal. Cardiovascular Imaging*.

[37] Gaur S, Øvrehus KA, Dey D, Leipsic J, Bøtker HE, Jensen JM (2016). Coronary plaque quantification and fractional flow reserve by coronary computed tomography angiography identify ischaemia-causing lesions. *European Heart Journal*.

[38] Driessen RS, Stuijfzand WJ, Raijmakers PG, Danad I, Min JK, Leipsic JA (2018). Effect of Plaque Burden and Morphology on Myocardial Blood Flow and Fractional Flow Reserve. *Journal of the American College of Cardiology*.

[39] Feuchtner GM, Barbieri F, Langer C, Beyer C, Widmann G, Friedrich GJ (2019). Non obstructive high-risk plaque but not calcified by coronary CTA, and the G-score predict ischemia. *Journal of Cardiovascular Computed Tomography*.

[40] Wu B, Pei X, Li ZY (2014). How does calcification influence plaque vulnerability? Insights from fatigue analysis. *TheScientificWorldJournal*.

