# Supplementary material for: Coronary Computed Tomography Angiography Atherosclerotic Plaque Volume as A Predictor of Myocardial Blood Flow Impairment in Non-Obstructive Coronary Artery Disease
Source: Rev Cardiovasc Med. 2025 Sep 26;26(9):39291. doi: 10.31083/RCM39291 (PMC12516756; doi:10.31083/RCM39291)
Supplement: Supplementary file 1 [file 2153-8174-26-9-39291-s1.docx]

**Supplementary Materials**

| **Table 1. Intra- and Inter-Observer Reproducibility of Quantitative CCTA Characteristics** | | | | |
| --- | --- | --- | --- | --- |
| **Quantitative CCTA Characteristics** | **Intra-observer reproducibility** | | **Inter-observer reproducibility** | |
|  | **ICC (95% CI)** | **Consistency** | **ICC, (95% CI)** | **Consistency** |
| Total plaque volume, mm3 | 0.81 (0.67-0.92) | good | 0.80 (0.66-0.91) | good |
| Total plaque burden, % | 0.82 (0.66-0.92) | good | 0.81 (0.65-0.91) | good |
| Non-calcified plaque volume, mm3 | 0.80 (0.65-0.92) | good | 0.78 (0.63-0.90) | good |
| Non-calcified plaque burden, % | 0.80 (0.63-0.92) | good | 0.78 (0.61-0.90) | good |
| Soft-tissue plaque volume, mm3 | 0.81 (0.63-0.92) | good | 0.79 (0.61-0.90) | good |
| Soft-tissue plaque burden, % | 0.81 (0.62-0.93) | good | 0.79 (0.60-0.89) | good |
| Fibrous plaque volume, mm3 | 0.81 (0.64-0.92) | good | 0.78 (0.62-0.90) | good |
| Fibrous plaque burden, % | 0.79 (0.62-0.91) | good | 0.77 (0.60-0.89) | good |
| Calcified plaque volume, mm3 | 0.97 (0.91-0.99) | excellent | 0.95 (0.89-0.97) | excellent |
| Calcified plaque burden, % | 0.94 (0.86-0.98) | excellent | 0.92 (0.84-0.96) | excellent |

Table footnotes: All data are presented as intraclass correlation coefficient with 95% confidence intervals, expressed as ICC (95% CI). CCTA, coronary computed tomography angiography.

| **Table 2. Univariate and Multivariate Logistic Regression Analysis of Predictors for Reduced Stress Myocardial Blood Flow (MFR <1.5 ml/min/g)** | | | | |
| --- | --- | --- | --- | --- |
| **Parameters** | **OR (95% CI)** | **p-value** | **OR (95% CI)** | **p-value** |
|  | **univariate analysis** | | **multivariate analysis** | |
| Age, years | 1.02 (0.96-1.09) | 0.50 | 0.97 (0.86-1.10) | 0.64 |
| Male sex, n (%) | 0.66 (0.21-2.15) | 0.49 | 1.50 (0.15-14.75) | 0.73 |
| Arterial hypertension, n (%) | 1.00 (0.99-1.01) | 0.99 | 0.95 (0.93-0.97) | 0.99 |
| Dyslipidemia, n (%) | 0.58 (0.18-1.82) | 0.35 |  |  |
| Current smoking, n (%) | 0.41 (0.11-1.54) | 0.19 | 0.48 (0.06-4.14) | 0.50 |
| BMI, kg/m^2^ | 1.05 (0.92-1.20) | 0.48 | 0.97 (0.78-1.20) | 0.78 |
| Type 2 diabetes mellitus, n (%) | 1.47 (0.09-25.03) | 0.79 | 1.40 (0.01-24.47) | 0.99 |
| EF, % | 0.95 (0.83-1.10) | 0.49 | 0.97 (0.82-1.17) | 0.78 |
| EDV, ml | 1.00 (0.98-1.03) | 0.75 |  |  |
| ESV, ml | 1.02 (0.98-1.06) | 0.32 |  |  |
| TC, mmol/l | 0.89 (0.56-1.43) | 0.64 |  |  |
| LDL-C, mmol/l | 0.87 (0.51-1.50) | 0.62 | 2.45 (0.23-25.88) | 0.46 |
| HDL-C, mmol/l | 1.25 (0.24-6.52) | 0.79 |  |  |
| TG, mmol/l | 0.98 (0.56-1.70) | 0.94 |  |  |
| Non-HDL-C, mmol/l | 0.89 (0.57-1.39) | 0.61 | 0.52 (0.07-4.14) | 0.54 |
| Fasting glucose, mmol/l | 0.63 (0.26-1.55) | 0.32 |  |  |
| eGFR, ml/min/1,73m^2^ | 0.98 (0.94-1.02) | 0.39 |  |  |
| CRP, mg/l | 1.10 (0.93-1.29) | 0.28 |  |  |
| Leukocytes, 10Е9/l | 1.04 (0.67-1.60) | 0.86 |  |  |
| Platelets, 10Е9/l | 1.00 (0.98-1.01) | 0.90 |  |  |
| Hemoglobin, g/l | 1.04 (0.99-1.09) | 0.11 |  |  |
| INR | 11.49 (0.10-13.20) | 0.31 |  |  |
| APTT, sec | 0.97 (0.86-1.09) | 0.63 |  |  |
| Fibrinogen, g/l | 1.02 (0.38-2.73) | 0.97 |  |  |
| Maximum stenosis, % | 1.02 (0.98-1.06) | 0.29 |  |  |
| Calcium Score, Agatston units | 1.00 (1.00-1.01) | 0.49 |  |  |
| Lumen, mm3 | 1.00 (1.00-1.00) | 0.09 |  |  |
| General volume, mm3 | 1.00 (1.00-1.00) | 0.054 |  |  |
| Total plaque volume, mm3 | 1.01 (1.00-1.02) | 0.006 | 1.01 (1.005-1.03) | <0.001 |
| Total plaque burden, % | 1.21 (1.05-1.40) | 0.009 |  |  |
| Non-calcified plaque volume, mm3 | 1.01 (CI 1.00-1.02) | 0.007 |  |  |
| Non-calcified plaque burden, % | 1.22 (1.04-1.43) | 0.01 |  |  |
| Soft-tissue plaque volume, mm3 | 1.07 (1.01-0.12) | 0.02 |  |  |
| Soft-tissue plaque burden, % | 2.53 (0.81-7.96) | 0.11 |  |  |
| Fibrous plaque volume, mm3 | 1.01 (1.00-1.02) | 0.008 |  |  |
| Fibrous plaque burden, % | 1.27 (1.06-1.52) | 0.009 |  |  |
| Calcified plaque volume, mm3 | 1.03 (0.10-1.06) | 0.09 |  |  |
| Calcified plaque burden, % | 1.59 (0.91-2.77) | 0.10 |  |  |

Table footnotes: All data are presented as odds ratios with 95% confidence intervals, expressed as OR (95% CI). APTT, activated partial thromboplastin time; BMI, body mass index; CRP, C-reactive protein; EDV, end diastolic volume; EF, ejection fraction; eGFR, estimated glomerular filtration rate; ESV, end systolic volume; HDL-C, high-density lipoprotein cholesterol; INR, international normalized ratio; LDL-C, low-density lipoprotein cholesterol; n, number of patients; Non-HDL-C, non-high-density lipoprotein cholesterol; TC, total cholesterol; TG, triglycerides.
